# The Effect of Systemic Racism and Homophobia on Police Enforcement and Sexual and Emotional Violence among Sex Workers in East London: Findings from a Cohort Study

**DOI:** 10.1007/s11524-022-00673-z

**Published:** 2022-10-12

**Authors:** Lucy Platt, Raven Bowen, Pippa Grenfell, Rachel Stuart, M. D. Sarker, Kathleen Hill, Josephine Walker, Xavier Javarez, Carolyn Henham, Sibongile Mtetwa, James Hargreaves, M.-C. Boily, Peter Vickerman, Paz Hernandez, Jocelyn Elmes

**Affiliations:** 1 Faculty of Public Health and Policy, London School of Hygiene and Tropical Medicine, London, UK; 2 National Ugly Mugs, Manchester, UK; 3 College of Business, Arts and Social Sciences, Brunel University, London, UK; 4 Faculty of Health and Life Sciences, Coventry University, Coventry, UK; 5 Population Health Sciences, Bristol Medical School, University of Bristol, Bristol, UK; 6 School of Health Care, University of Leeds, Leeds, UK; 7 Department of Infectious Disease Epidemiology, Imperial College, London, UK; 8 Open Doors, Homerton University Foundation Trust, London, UK

**Keywords:** Sex work, Ethnicity, Sexuality, Emotional and sexual violence, Policing

## Abstract

There is extensive qualitative evidence of violence and enforcement impacting sex workers who are ethnically or racially minoritized, and gender or sexual minority sex workers, but there is little quantitative evidence. Baseline and follow-up data were collected among 288 sex workers of diverse genders (cis/transgender women and men and non-binary people) in London (2018–2019). Interviewer-administered and self-completed questionnaires included reports of rape, emotional violence, and (un)lawful police encounters. We used generalized estimating equation models (Stata vs 16.1) to measure associations between (i) ethnic/racial identity (Black, Asian, mixed or multiple vs White) and recent (6 months) or past police enforcement and (ii) ethnic/racial and sexual identity (lesbian, gay or bisexual (LGB) vs. heterosexual) with recent rape and emotional violence (there was insufficient data to examine  the association with transgender/non-binary identities). Ethnically/racially minoritized sex workers (26.4%) reported more police encounters partly due to increased representation in street settings (51.4% vs 30.7% off-street, *p* = 0.002). After accounting for street setting, ethnically/racially minoritized sex workers had higher odds of recent arrest (adjusted odds ratio 2.8, 95% CI 1.3–5.8), past imprisonment (aOR 2.3, 95% CI 1.1–5.0), police extortion (aOR 3.3, 95% CI 1.4–7.8), and rape (aOR 3.6, 95% CI 1.1–11.5). LGB-identifying sex workers (55.4%) were more vulnerable to rape (aOR 2.4, 95% CI 1.1–5.2) and emotional violence. Sex workers identifying as ethnically/racially minoritized (aOR 2.1, 95% CI 1.0–4.5), LGB (aOR 2.0, 95% CI 1.0–4.0), or who use drugs (aOR 2.0, 95% CI 1.1–3.8) were more likely to have experienced emotional violence than white-identifying, heterosexual or those who did not use drugs. Experience of any recent police enforcement was associated with increased odds of rape (aOR 3.6, 95% CI 1.3–8.4) and emotional violence (aOR 4.9, 95% CI 1.8–13.0). Findings show how police enforcement disproportionately targets ethnically/racially minoritized sex workers and contributes to increased risk of rape and emotional violence, which is elevated among sexual and ethnically/racially minoritized workers.

## Background

Review evidence suggests that between 18 and 44% of sex workers providing in-person services report physical violence at work in the last year, between 24–54% depression and 5–58% anxiety [1, 2]. This masks extensive diversity within sex-working communities as experience of violence and mental health is shaped by the legal context and structural inequalities in access to housing, health and social services, drug use, adequacy of income support, and the quality of working environments [2–4]. Inequalities are compounded by criminalization: police enforcement often results in an immediate loss of income, necessitating longer working hours and compromises in pricing, client selection, sexual services, health, and safety [5]. There is an emerging evidence base documenting how repressive policing and poorer health experienced by sex workers is also fuelled by racism, homophobia, and transphobia [5]. Qualitative research shows that sex workers from racialized, sexual and gender minority groups experience more intensive police enforcement as well as physical and sexual violence from police and other perpetrators [6–8]. Evidence from Canada shows how the legacy of colonial and racist policies contributes to increased marginalization of sex workers identifying as indigenous, with higher prevalence of HIV, greater representation in street-based settings, frequent intergenerational sex work, and having children taken into care [9–11].

More broadly across the population, ethnic and racialized minorities experience extensive health inequalities and injustices linked to structural racism [12, 13]. Racial discrimination experienced within health care or society can influence how people engage with health services as well as quality of health care access received [13]. Discrimination towards Black and people of color as well as Gypsy, Roma, or Travelers results in poorer mental health outcomes, particularly when experienced as physical violence and verbal abuse from police and the broader community [14–16]. There is clear evidence of racism within criminal justice systems. The use of more frequent and excessive force is evidenced by disproportionate fatal police shootings against Black, Indigenous, and people of color in the USA [15, 17]. In England and Wales, Black and Asian minorities are over-represented throughout the criminal justice system: at the point of stop and search; arrests; custodial sentencing; deaths in police custody; and in the prison population, where 27% identify as Black or Asian, relative to 13% of the general population [18]. Similar injustices are observed among Gypsy and Traveler communities who in 2019 made up 6–7% of the prison population (men and women) in comparison to 0.1% of the population as a whole [19]. Institutional homophobia and transphobia can be overt in the form of colonial-era laws criminalizing gender non-conformity and homosexuality in some settings or indirectly manifested in the form of disproportionate incarceration rates among gender and sexual minorities [20, 21]. Similarly, discrimination, fear, hostility, and violence towards trans, lesbian, gay, and bisexual communities have also been evidenced to contribute to minority stress, reduced access to health services, increased sexual and physical violence, and poorer mental health [22].

Existing analytical tools such as the risk environment framework help characterize how structural factors (e.g., law, gender or racial discrimination), workplace conditions (e.g., settings), and interpersonal/individual behaviors (e.g., frequent heroin or crack use) shape health inequalities among sex workers and intersect with sex work criminalization [5, 23, 24]. Intersectionality theory argues that multiple forms of oppression such as those experienced by Black women, sexual and gender minorities are mutually constitutive and interdependent [25]. Stigma and discrimination across the risk environment produce distinct experiences of health care services among women living with HIV depending on their intersecting racial, sexual, gender, and sex worker identities [26]. This can also be seen at a macro-structural level, where interacting contexts produced by multiple forms of criminalization (e.g., of sex work, homosexuality, drugs or anti-immigration laws) and institutional discrimination reduces access to services and increases police violence, disproportionately impacting racially minoritized and marginalized communities [26–29]. Research suggests that ethnic or racialized minorities make up a substantial proportion of the sex worker community (9.4–22.8% in the UK and the USA) and up to 18.6% of sex workers identify as LGB [7, 30–32]. Less is known about gender diverse sex workers, but estimates from the UK suggest that between 1 and 4% of sex workers identify as transgender [33]. Yet despite this, there has been little quantitative research on the extent to which oppression and discrimination at the intersections of race, gender, and sexual minority identity among sex workers may increase instances of violence and repressive policing. Drawing on data from a prospective cohort study of cis and trans female, male, and non-binary sex workers in London, we examine the extent to which police enforcement practices differ by ethnic or racial identity. It was not possible to look at the association between transgender or gender diverse identity and policing due to the small sample size. We also examined the extent to which racialized and sexual minorities are at risk of recent emotional violence or rape.

## Methods

This research was participatory. People with experience of sex work or of working with sex workers, and researchers, co-developed the overarching research question, data collection, analyses, and write up. Methods and key findings from the analyses among cis and trans women have been published previously [34]. Between May 2018 and October 2019, sex workers of all genders were enrolled in a prospective open cohort with two rounds of data collection at baseline and 6-month follow-up. Sex workers who had exchanged in-person sexual services in the last 3 months in East London (or other parts of London for sex workers advertising online), aged ≥ 18 years, were invited to complete a questionnaire. We used time-location sampling of sex workers working on the street and targeted sampling of online profiles. We supplemented recruitment with convenience sampling (e.g., in NHS-clinics, snowball sampling) and expanded baseline recruitment for street-based sex workers.

Participants self-completed a questionnaire on a tablet or online using Open Data Kit software (Open Data Kit version 1.28.4) or read out by interviewers when support was needed. Data were collected on the following: socio-demographic characteristics; sex work organization; access to health and social services; mental health; policing; sexual, physical, and emotional violence from clients, intimate partners, police, and other perpetrators (including residents, strangers, drug dealers). Indicators were drawn from validated measures or developed through findings from a linked qualitative study [29]. The questionnaire was available in English, Romanian, Polish, and Portuguese and took 60 min to complete. Three follow-up attempts of the original baseline sample were made by phone and email, supplemented with street outreach. All participants were offered £20 at baseline data collection as reimbursement. An increased rate of £40 was given at follow-up, in recognition as a more appropriate compensation for working time lost. The study was approved by the London School of Hygiene and Tropical Medicine’s and the NHS Stanmore’s research ethics committee (IRAS ID 231,206).

### Outcome Measures

Exposure to direct law enforcement was assessed at baseline and follow-up and included the following: arrest; client arrest; sanctions (cautions, warnings, notices); valuables, condom, drugs or drug paraphernalia confiscation; referral to services; or imprisonment; in the previous 6 months or ever. A single combined variable included all items of recent law enforcement. Unlawful police practices included the following: extortion (police accepted sex or money or other goods in lieu of arrest or to avoid trouble); verbal abuse (being belittled, humiliated or had abusive or insulting language such as racist remarks directed at you); being scared (scared or intimidated, made sexual comments); raped (forced anal, vaginal, oral sex); personal property been damaged; threatened; or actual physical violence. A single combined variable included all items of verbal, physical, or sexual assault by police. Measures of sexual or emotional violence from intimate partners, clients, or others included the following: (i) recent (last 6 months) experience of rape (forced oral, vaginal or anal sex without consent) and (ii) recent emotional violence (being belittled or humiliated or had abusive or insulting language directed towards you such as calling you inappropriate names or making racist remarks, being scared or intimidated).

### Explanatory Variables

Ethnic or racial identity was categorized as a binary variable comparing the experiences of those self-identifying as African, Arab, Asian, Asian British, Black, Black British, Caribbean, Middle Eastern, mixed or multiple ethnic groups, Roma, Traveler with white English, Welsh, Scottish, Irish, European, or other sex workers. Sexual identity was categorized as a binary variable by self-identification as lesbian/gay/homosexual or bisexual or straight/heterosexual. We grouped self-identified gender identity into male (including cis and trans male) and female (including cis and trans female). There were too few trans/non-binary participants to examine their experiences separately. For non-binary participants, we examined the gender identity they worked under (which was in all cases cis or trans female or male). We asked about recent (last 6 months) circumstances of sex work including the following: working sector (meeting clients in outdoors settings in past 6 months vs indoors or online); working alone vs working with others; screening of clients (never/sometimes vs always); and time spent in sex work. We categorized those who had not used drugs in the last 4 weeks as non-current drug users and increasing risk related to alcohol use as ≥ 5 on the AUDIT-C scale. Indicators of social exclusion included finding it difficult to meet usual expenses, recent (last 6 months) eviction, and current (last 4 weeks) homelessness, defined as sleeping rough or living in unstable accommodation (e.g., parent’s or friend’s home, sheltered, or homeless accommodation). Migration status was defined in two ways: UK national/permanent residence/indefinite leave to remain versus overseas national/refugee/asylum seek/unknown or being born in the UK versus not. We measured mental health through a validated single composite measure (PHQ-4) with a cut-point of ≥ 6 to indicate symptoms of depression or anxiety [34, 35].

### Analyses

We examined differences in socio-demographic characteristics, circumstances of sex work, violence, mental health and access to services by ethnic/racial identity at baseline, using Pearson *χ*^2^ tests (categorical variables) and two-sample Wilcoxon Mann–Whitney tests (continuous variables). We assessed associations between ethnic/racial identity and recent or past police enforcement practices adjusting for potential confounders. We examined univariable and multivariable associations between ethnic/racial and sexual identity with the following: (i) recent (last 6 months) rape and (ii) recent emotional violence. We used generalized estimating equation (GEE) logistic regression with an exchangeable correlation matrix in all analyses because the factors potentially associated with violence and police enforcement during follow-up were serial (time dependent) measures. These models also account for the correlation of repeated observations on some participants, so that analyses included all participants irrespective of follow-up. We considered working sector, gender, migration status, and duration in sex work as a priori confounders in all analyses [1, 2, 5, 34]. For sexual and emotional violence outcomes, we also examined the following: current drug use; unstable housing in the form of current homelessness or recent eviction; any recent law enforcement; circumstances of sex work; and difficulty meeting usual expenses as potential confounders. We report unadjusted and adjusted odds ratios (OR) and 95% confidence intervals (CI) of the associations.

## Results

**Fig. 1:**
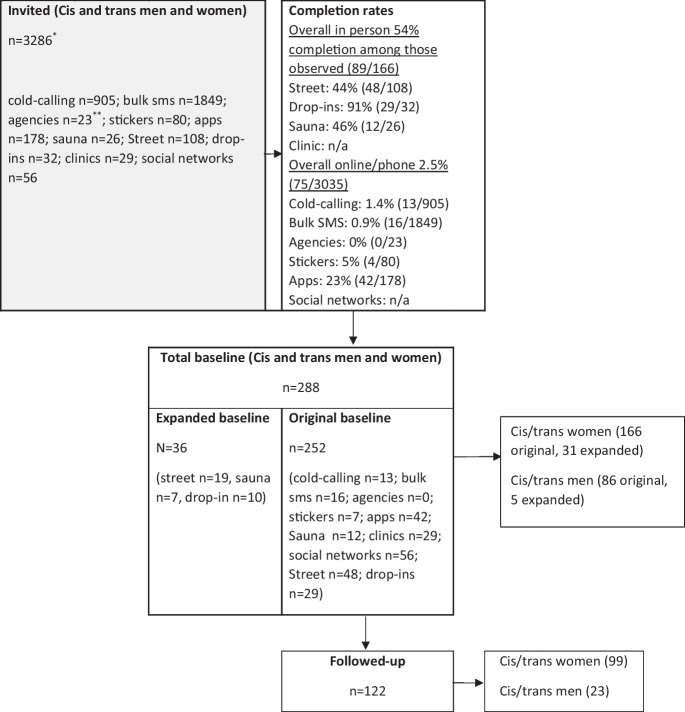
Flowchart of individuals who completed baseline surveys

A total of 288 individuals completed baseline surveys (252 original and 36 expanded baseline) (Fig. 1). A total of 274 individuals reported an ethnic/racial identity: 26.4% (*n* = 72) identified as an ethnic/racialized minority including Asian, Asian British (*n* = 12, 4.4%), Black/African/Caribbean/Black British (*n* = 25, 9.1%), mixed or multiple ethnicities (*n* = 22, 8.0%), or otherwise ethnically or racially minoritized (*n* = 13, 4.7%) including Traveler or Roma (< 10) and Middle Eastern (< 5). Overall, 73.7% (*n* = 202) identified as white including British (*n* = 100, 36.5%), Irish (*n* = 8, 2.9%), or European (*n* = 86, 31.4%) and other or unknown (*n* = 8, 2.9%). Among the sample, 197 (72.2%) identified as female and 76 (27.8%) as male. Within this, fewer than ten individuals overall identified as trans women, trans men, or non-binary. Overall, 55% (*n* = 143) identified as LGB, 92.4% among cis/trans men, and 41.7% among cis/trans women. In total, 35.9% (*n* = 103) found clients from street settings, and 34.1% from flats, saunas, or through online advertising, and the majority (60.8%) worked alone. Just under half (47% *n* = 137) were born outside the UK, and less than a third (28.5% *n* = 78) were not UK nationals. The median age was 31 years (IQR = 25–39 years), and the median duration in sex work was 6 years (IQR = 3–14 years). Overall, 122 individuals were followed up (retention 58.6%). Retention did not differ by ethnic or racial identity or sector but did differ by gender. Retention was 59.6% (99/169) among cis/trans women and 29.2% (21/72) among cis/trans men (Table 1).

**Table 1:** Characteristics of all participants stratified by ethnic or racial identity at baseline

		Ethnic or racial identity		*p* value*
	All	Ethnically minoritized	White-identity	
	*n* (%)	*n* (%)	*n* (%)	
Total	274^#^	72 (26.4%)^§^	202 (73.7%)	
Demographic characteristics				
Gay, lesbian, homosexual or bisexual⌃	143 (55.4%)	34 (49.3%)	109 (57.7%)	0.230
Cis or trans man**	76 (27. 8%)	19 (27.1%)	55 (27.8%)	0.919
Overseas national	78 (28.5%)	12 (16.9%)	65 (32.7%)	0.012
Born outside the UK	137 (47.7%)	35 (48.6%)	92 (45.5%)	0.654
Education (primary/secondary)	130 (46.9%)	34 (48.6%)	91 (46.4%)	0.758
Age (median years (IQR))	31 (25–39)	33 (27–47)	30 (18–52)	0.007
Circumstances of sex work				
Street as main place to find clients	103 (35.9%)	37 (51.4%)	62 (30.7%)	0.002
Median years in sex work (IQR)	6 (3–14)	8 (3–21)	5 (2–12)	< 0.001
Age of first sex work (median) (IQR))	22 (18–27)	21 (18–27)	22 (19–27)	0.318
Number of clients a week (> 3)	135 (47.4%)	33 (45.8%)	97 (48.0%)	0.750
Mostly work alone	155 (60.8%)	44 (59.1%)	104 (63.8%)	0.501
Mostly work at night	127 (51.4%)	33 (52.4%)	89 (51.4%)	0.899
Sex work as main income	196 (70.5%)	50 (69.4%)	137 (71.0%)	0.807
Always works with CCTV camera	55 (24.9%)	17 (27.9%)	37 (24.3%)	0.638
Always screen clients	110 (44.2%)	28 (41.2%)	80 (46.8%)	0.432
Indicators of social exclusion				
Difficulty in making ends meet	174 (60.6%)	49 (68.1%)	116 (57.8%)	0.114
Currently in arrears	122 (53.5%)	29 (49.2%)	90 (55.6%)	0.398
Not receiving benefits	161 (66.0%)	47 (72.3%)	110 (64.0%)	0.225
In unstable housing (in the last 4 weeks)	78 (28.6%)	25 (36.8%)	49 (25.0%)	0.095
Past eviction	67 (25.9%)	17 (25.8%)	47 (26.0%)	0.902
Children taken into care at some point	32 (12.1%)	7 (9.7%)	25 (12.4%)	0.530
Access to services				
Registered at a GP practice	197 (68.6%)	58 (80.6%)	132 (65.3%)	0.016
Recently accessed sex worker project	95 (33.1%)	30 (41.7%)	61 (30.4%)	0.076
Unmet mental health need***	90 (34.5%)	33 (47.8%)	53 (28.9%)	0. 005
Alcohol and other drug use				
Problematic drinking (Audit-C ≥ 5)	115 (43.1%)	26 (38.2%)	87 (46.3%)	0.252
Current drug use (4 weeks)^$^	159 (59.6%)	49 (70.0%)	104 (55.3%)	0.033
Daily crack or heroin use	70 (24.4%)	25 (34.7%)	43 (21.3%)	0.023
Ever injected drugs	58 (21.5%)	19 (28.4%)	37 (19.1%)	0.110
Partner supplies drugs	70 (26.8%)	27 (39.7%)	40 (21.6%)	0.007
Violence and mental health				
Anxiety/depression (PHQ4)	122 (42.5%)	30 (41.7%)	86 (42.6%)	0.894
Recent rape (6 months)	52 (21.9%)	16 (25.4%)	34 (20.7%)	0.448
Recent emotional violence (6 months)	191 (67.0%)	59 (83.1%)	126 (62.4%)	0.001

^*^p value derived from Pearson χ^2^ tests (categorical variables) and two-sample Wilcoxon Mann–Whitney tests (continuous variables)

^#^Fourteen people did not report an ethnic identity

⌃Gay/homosexual (*n* = 53), bisexual (*n* = 71), any other term or do not use a term (*n* = 9)

^**^Trans men, trans women (*n* =  < 10)

^***^Wanted mental health treatment, had not received it

^$^Eighty-eight (55%) use heroin or crack; 71 used ecstasy, amphetamines, marijuana, cocaine

^§^Asian/Asian British (*n* = 12), Black, African, Caribbean, Black British (*n* = 25), multiple, mixed, other (including Hispanic, Latino, other Asian) (*n* = 22), traveler or Roma (< 10), Middle Eastern (< 5)

### Characteristics of Sex Workers by Ethnic and Racial Identity

Overall, proportionally, more ethnically/racially minoritized sex workers worked in street-based settings (*n* = 37, 51.4%) compared to white-identifying sex workers (*n* = 62, 30.7%). They were also older (33 vs. 30 years) and more likely to be UK nationals, but there was no difference in terms of gender or sexual identity or educational attainment. Other than work setting, most aspects of sex work organization were comparable, although on average ethnically/racially minoritized participants worked in sex work longer (8 vs 5 years). Proportionally, more ethnically/racially minoritized participants were homeless or in temporary accommodation (37.7% vs 25.0%) — which is also more frequent for street-based sex workers [34] — but there was no difference in past eviction (26%). Just under half the sample (53.5%, *n* = 122) reported being in arrears, two thirds did not receive state benefits (*n* = 174), and this did not differ by ethnic/racial identity.

Ethnically or racially minoritized participants reported higher prevalence of recent drug use (70.0% vs 55.3%) overall, which is again more frequent among street-based sex workers [34]. Key differences were recorded in daily heroin or crack use (34.7% vs 21.3%), partners supplying drugs (39.7 vs 21.6%), and a history of injecting drug use (28.4% vs 19.1%). Drinking alcohol at levels of increasing risk (≥ 5 on Audit-C) was proportionally lower among ethnically or racially minoritized compared to white-identifying participants (38.2% vs 46.3%).

Proportionally, more ethnically or racially minoritized participants were registered at GPs and accessed specialist sex worker services. A third of the sample reported being denied access to a mental health service when in need and this was higher among ethnically/racially minoritized sex workers (47.8% vs 28.9%). Prevalence of symptoms of anxiety or depression was 42.5% (*n* = 122) overall, and 21.9% (*n* = 52) of participants reported being raped in the last 6 months, with no difference by ethnic/racial identity. Proportionally, more ethnically or racially minoritized sex workers reported experiencing emotional violence in the last 6 months (83.1 vs 62.4%).

### The Effect of Ethnic/Racial Identity on Experiences of Police Enforcement

Overall, 18.6% (*n* = 49) of the sample had been arrested in the last 6 months for any reason and 6.8% for sex work. Just over a quarter (26.5%, *n* = 72) had ever been in prison. Across virtually all unadjusted measures, proportionally more sex workers identifying as ethnically/racially minoritized had been exposed to enforcement compared to white-identifying sex workers. After accounting for working sector, as well as gender, sexual identity, country of birth, and duration of time in sex work, odds of being arrested recently remained significantly higher for ethnically/racially minoritized sex workers (adjusted odds ratio 2.8, 95% CI 1.3–5.8). Odds of past imprisonment also remained higher for this population (aOR 2.3, 95% CI 1.1–5.0).

Among harmful police practices, overall, 32.1% (*n* = 89) had ever experienced any police violence; 7.0% (*n* = 18) of participants had experienced extortion; 13.2% (*n* = 34) had property damaged; 23.5% (*n* = 61) verbal abuse; and 20.1% had been scared (*n* = 52) and 4.2% (*n* = 11) had been raped by police officers. Following adjustment, ethnically/racially minoritized participants were more likely to have experienced extortion (aOR 3.3, 95% CI 1.4–7.8) and rape by police (aOR 3.6, 95% CI 1.1–11.5). There was no difference in the levels of other measures of harmful police practices. These findings are summarized in Table 2.

**Table 2:** Prevalence of lawful and unlawful police enforcement practices among all sex workers and associations with ethnic or racial identity

	Ethnic/Racial identity			Odds ratio for ethnically/racially minoritized identity			
	All	Ethnically minoritized	White	Unadjusted		Adjusted*	
Police enforcement practice	*n*/total (%)	*n* (%)	*n* (%)	OR (95% CI)	*p* value	OR (95% CI)	*p* value
Recent (6 months) practices							
Arrested (any reason)	49/264 (18.6%)	22 (31.4%)	24 (13.2%)	3.0 (1.6–5.4)	0.001	2.8 (1.3–5.8)	0.008
Arrested (sex work)	18/264 (6.8%)	4 (5.9%)	14 (7.6%)	0.7 (0.3–2.1)	0.554	0.4 (0.1–1.4)	0.141
Client arrested by the police	32/273 (11.7%)	10 (14.9%)	21 (10.8%)	1.3 (0.6–2.7)	0.453	0.6 (0.2–1.5)	0.262
Sanctioned by police	74/276 (26.8%)	28 (39.4%)	42 (21.8%)	2.2 (1.3–3.7)	0.003	1.6 (0.8–3.2)	0.201
Recently been to prison	18/272 (6.6%)	7 (9.9%)	10 (5.3%)	1.8 (0.7–4.1)	0.203	1.3 (0.5–3.3)	0.552
Referred to services by police	21/265 (7.9%)	8 (11.4%)	11(6.0%)	1.9 (0.9–4.2)	0.111	1.0 (0.4–2.5)	0.929
Items confiscated by police	40/279 (14.3%)	15 (21.1%)	22 (11.2%)	2.4 (1.6–4.6)	0.009	1.5 (0.7–3.4)	0.31
Any law enforcement	103/283 (36.4%)	38 (53.5%)	60 (30.0%)	2.4 (1.5–3.4)	0.001	1.5 (0.8–3.61)	0.242
Past (ever) practices							
Arrested (any reason)	113/265 (42.6%)	38 (54.3%)	70 (38.3%)	1.7 (1.1–2.6)	0.016	1.4 (0.6–3.1)	0.422
Arrested (sex work)	51/265 (19.2%)	14 (20.6%)	36 (19.5%)	1.2 (0.6–2.1)	0.602	0.6 (0.3–1.3)	0.183
Client ever arrested by police	57/274 (20.8%)	22 (32.4%)	34 (17.4%)	1.9 (1.1–3.4)	0.022	1.0 (0.5–2.3)	0.91
Ever been sanctioned by police	123/272 (45.2%)	41 (57.7%)	77 (40.7%)	1.9 (1.2–3.0)	0.006	1.5 (0.7–3.3)	0.327
Ever had items confiscated by police	74/275 (26.9%)	23 (32.4%)	46 (24.0%)	1.5 (0.9–2.6)	0.132	1.0 (0.5–1.9)	0.924
Ever been to prison	72/272 (26.5%)	30 (42.3%)	38 (20.1%)	2.4 (1.4–3.9)	< 0.001	2.3 (1.1–5.0)	0.037
Ever been referred to services	43/260 (16.5%)	13 (18.6%)	28 (15.7%)	1.5 (0.8–2.7)	0.18	0.9 (0.4–1.8)	0.224
Ever experienced any law enforcement	123/280 (43.9%)	39 (54.9%)	79 (40.1%)	1.8 (1.1–2.9)	0.016	1.0 (0.5–2.0)	0.904
Past unlawful police practices							
Accepted money, sex in lieu of arrest	18/258 (7.0%)	11 (15.5%)	7 (4.0%)	4.0 (1.7–9.8)	0.002	3.3 (1.4–7.8)	0.008
Verbal abuse	61/260 (23.5%)	19 (27.1%)	40 (22.2%)	1.3 (0.7–2.3)	0.419	0.8 (0.4–1.4)	0.408
Scaring or intimated	52/259 (20.1%)	18 (25.7%)	32 (18.0%)	1.4 (0.8–2.9)	0.134	1.4 (0.7–2.8)	0.304
Rape (anal, vaginal or oral)	11/262 (4.2%)	6 (8.7%)	5 (2.8%)	4.3 (1.4–13.5)	0.012	3.6 (1.1–11.5)	0.032
Threatened or actual physical violence	32/264 (12.1%)	13 (18.8%)	18 (9.8%)	1.9 (1.0.–4.0)	0.053	1.8 (0.9–3.5)	0.112
Damaged personal property	34/258 (13.2%)	17 (24.3%)	17 (9.6%)	2.3 (1.2–4.4)	0.012	1.9 (0.9–3.7)	0.082
Any police violence ever	89/277 (32.1%)	29 (40.8%)	57 (29.2%)	1.5 (0.9–2.5)	0.128	1.1 (0.6–2.0)	0.702

^*^Univariable and multivariable models are from generalized estimating equation (GEE) logistic regression with an exchangeable correlation matrix. Multivariable models are adjusted for working sector, time in sex work, born in the UK or not, gender, and sexual identity

### The Effect of Ethnic/Racial and Sexual Identity on Rape and Emotional Violence

#### Experiences of Rape

LGB-identifying sex workers were more likely to have been raped by any perpetrator (aOR 2.4, 95% CI 1.1–5.2), but there was no evidence of differences by ethnic/racial identity (aOR 0.9, 95% CI 0.4–1.9). Other factors significantly associated with having been raped included recent law enforcement (aOR 3.6, 95% CI 1.3–8.4), current drug use (aOR 3.8, 95% CI 1.1–10.1), recent eviction (aOR 3.1, 95% CI 1.1–8.4), and not meeting daily expenses (aOR 2.3, 95% CI 1.1–4.5). These effects were significant irrespective of gender identity, working sector or time in sex work.

#### Emotional Violence

Sex workers identifying as ethnic/racialized minorities were more likely to have experienced recent emotional violence (aOR 2.1, 95% CI 1.0–4.5) compared to white-identifying sex workers, as were those currently using drugs (aOR 2.0, 95% CI 1.1–3.8). There was also evidence that sex workers identifying as LGB were at increased risk of emotional violence (aOR 2.0, 95% CI 1.0–4.0). Sex workers who had experienced any recent law enforcement (aOR 4.9, 95% CI 1.8–13.0) were more likely to have experienced emotional violence than those who had not. Cis or trans men were less likely to have experienced emotional violence (aOR 0.2, 95% CI 0.1–0.5) compared with cis or trans women, as were people working in sex work longer than 15 years (aOR 0.5, 95% CI 0.3–1.0) and those who had only attended school up to secondary level (aOR 0.4, 95% CI 0.2–0.7) (Table 3).

**Table 3:** Unadjusted and adjusted factors associated with recent rape and emotional violence

	Rape in the last 6 months				Emotional violence in the last 6 months			
			Unadjusted	Adjusted*			Unadjusted	Adjusted**
	*n*/*N*	%	OR (95% CI)	OR (95% CI)	*n*/*N*	%	OR (95% CI)	OR (95% CI)
Ethnicity/race								
White	34/164 (20.7%)		1.0	1.0	126/202 (62.4%)		1.0	1.0
Ethnic/racialized minority	16/63 (25.4%)		1.4 (0.8–2.6)	0.9 (0.4–1.9)	59/71 (83.1%)		2.2 (1.2–3.9)	2.1 (1.0–4.5)
Sexual identity								
Heterosexual	20/108 (18.5%)		1.0	1.0	86/117 (72.5%)		1.0	1.0
LGB	28/112 (25.0%)		1.2 (0.7–2.2)	2.4 (1.1–5.2)	94/143 (65.7%)		0.7 (0.4–1.1)	2.0 (1.0–4.0)
Gender identity								
Cis/trans women	41/179 (22.9%)		1.0	1.0	152/197 (77.2%)		1.0	1.0
Cis/trans men	8/50 (16.0%)		0.7 (0.3–1.4)	1.3 (0.5–3.4)	33/76 (43.3%)		0.3 (0.2–0.4)	0.2 (0.1–0.5)
Migration status								
UK citizen	43/164 (26.2%)		1.0		136/196 (69.4%)		1.0	
Non–UK citizen	7/65 (10.8%)		0.3 (0.1–0.7)	NS	48/77 (62.3%)		0.8 (0.5–1.3)	NS
Education								
Higher education	24/120 (20.0%)		1.0		97/147 (66.0%)		1.0	1.0
Secondary/primary	24/110 (21.8%)		1.3 (0.7–2.2)	NS	87/129 (67.4%)		0.8 (0.5–1.2)	0.4 (0.2–0.7)
Drug use last 4 weeks								
No	8/88 (9.1%)		1.0	1.0	53/108 (49.1%)		1.0	1.0
Yes	44/141 (31.2%)		4.9 (2.2–10.8)	3.8 (1.1–10.1)	133/159 (83.6%)		3.9 (2.4–6.3)	2.0 (1.1–3.8)
Sex work sector								
Off-street work	32/90 (35.6%)		1.0	1.0	102/182 (56.0%)		1.0	1.0
Street-based	20/147 (13.6%)		4.9 (2.7–8.9)	1.3 (0.5–3.7)	89/103 (86.4%)		4.1 (2.4–6.9)	1.3 (0.6–3.2)
Time in sex work								
< 15 years	26/125 (20.8%)		1.0	1.0	101/145 (69.7%)		1.0	1.0
> 15 years	25/109 (22.9%)		1.1 (0.6–1.9)	0.9 (0.5–1.8)	89/135 (65.9%)		0.7 (0.5–1.1)	0.5 (0.3–1.0)
Work arrangements								
With others	16/85 (18.8%)		1.0		68/100 (68.0%)		1.0	
Alone	33/130 (25.4%)		1.2 (0.7–2.2)	NS	112/155 (72.3%)		1.4 (0.9–2.2)	NS
Screening clients								
Never	29/118 (24.6%)		1.0		99/139 (71.2%)		1.0	
Always	19/94 (20.2%)		0.7 (0.4–1.2)	NS	79/110 (71.8%)		0.9 (0.6–1.4)	NS
Current housing								
No	23/161 (14.3%)		1.0		122/195 (62.6%)		1.0	1.0
Yes	29/73 (39.7%)		3.4 (2.0–5.8)	–	65/78 (83.3%)		2.4 (1.5–3.8)	1.3 (0.6–2.8)
Recent eviction								
No	41/213 (19.2%)		1.0	1.0	169/240 (70.4%)		1.0	
Yes	8/14 (57.1%)		6.3 (2.5–15.6)	3.1 (1.1–8.4)	14/15 (93.3%)		5.5 (1.63–18.4)	-
Recent enforcement								
No	16/148 (10.8%)		1.0	1.0	99/180 (55.0%)		1.0	1.0
Yes	36/89 (40.4%)		6.1 (3.4–11.0)	3.6 (1.3–8.4)	92/103 (89.3%)		6.0 (3.3–11.0)	4.9 (1.8–13.0)
Meet daily expenses								
No	13/95 (13.7%)		1.0	1.0	66/112 (58.9%)		1.0	
Yes	39/142 (27.5%)		2.80(1.5–5.2)	2.3 (1.1–4.5)	125/173 (72.3%)		1.7 (1.1–2.7)	NS

–, only one indicator of unstable housing was adjusted for in the final model

*NS* non-significant

^*^GEE model adjusted for ethnic and sexual identity, gender, citizenship status, drug use in the last 4 weeks, sex work sector, duration in sex work, recent eviction, any recent law enforcement, ability to meet daily expenses

^**^Adjusted for ethnic and sexual identity, gender, education, drug use in the last 4 weeks, sex work sector, duration in sex work, current housing status, any recent law enforcement

## Discussion

This is the first study in the UK to highlight the extent to which ethnically or racially minoritized sex workers experience disproportionate police enforcement and to quantify more broadly differences in the experiences of violence by ethnic/racial and sexual identity. Among participants, ethnically or racially minoritized sex workers more frequently worked from street settings and experienced more homelessness, crack, or heroin use and police enforcement. After accounting for street-based work and drug use, they continued to experience disproportionate arrest, imprisonment, extortion, and rape from police. Ethnically or racially minoritized participants were more likely to have experienced emotional violence in the form of abuse and harassment from clients, intimate partners, or other members of the community, and this emotional violence was pervasive across the sample. They were also more likely to have been denied access to mental health services when in need. One in five participants had been raped in the last 6 months, and this was even more likely for LGB participants, irrespective of work sector or drug use.

Findings corroborate other research that illustrates disparities within sex working communities and add evidence of the manifestations of structural racism [36, 37]. Previous evidence demonstrates that ethnically or racially minoritized sex workers are more vulnerable to police enforcement, homelessness, and social exclusion in relation to drug use, through their greater representation in street-based settings [10, 34], yet our research demonstrates that after accounting for these vulnerabilities, odds of arrest, imprisonment, police extortion, or rape remain higher among sex workers identifying as ethnically/racially minoritized. Our findings provide further evidence showing how criminalization of sex work not only is ineffective at protecting sex workers but also increases their vulnerability to violence and exacerbates marginalization of ethnically and racially minoritized groups [5, 30]. Our finding of higher levels of emotional violence, which include racist insults, experienced by ethnically or racially minoritized sex workers, alongside greater likelihood of being denied access to mental healthcare, supports previous evidence that intersecting racism and sex work stigma is linked to worse health outcomes [26, 38].

While there was some evidence that ethnically/racially minoritized participants were more likely to have been raped by police — an important finding itself — we did not identify evidence for any difference for recent rape by clients, intimate partner or others. Other evidence from a comparable sample in Baltimore, USA, found sex workers identifying as Black to have reduced risk of sexual/physical violence from clients compared to White-identifying counterparts, attributed to less frequent drug use or working indoors [30]. Our finding of an association between current drug use and elevated odds of recent rape supports this, alongside previous analyses showing far higher levels of all types of violence perpetrated against cis/trans female sex workers working from street settings than indoors [34]. While we found higher levels of current drug use, including daily heroin or crack use, among ethnically/racially minoritized participants, this is due to their increased representation in street-based settings — where 91% report daily crack or heroin use [34]. Sex workers dependent on crack or heroin are more often targeted by violent perpetrators, and this, combined with the high cost and criminalized context in which these drugs are sold, is a key driver of street-based sex work and social exclusion [34, 39, 40]. We find that sex workers identifying as LGB are at increased risk of both rape from any perpetrator and emotional violence, supporting other research showing the increased vulnerability of the LGB community, and LGB sex workers in particular, to violence [7, 27]. Recent rape is also associated with unstable housing. LGB and young people from ethnically/racially minoritized groups disproportionately experience homelessness in the USA and UK, and this has been linked to increased violence [41, 42]. While current homelessness was no higher among LGB compared to heterosexual-identifying participants (22.6% vs 37%), it was high overall (29%) and particularly among those working in street settings (58.8%) [34] highlighting the intersecting harms of structural racism, homophobia, and related social exclusion.

A substantial proportion of cis/trans women in our sample identified as LGB (41.7%), far higher than among a general population survey in England and Wales (2.4%) [43]. The proportion of participants identifying as ethnically/racially minoritized in our sample (26.4%) is comparable with other studies among sex workers in London (26.9%) but lower than in our study boroughs in London (over 50%) [31]. We found discrepancies within ethnically/racially minoritized participants: with greater proportions of participants identifying as Black or Black British (34%) compared to the population in study boroughs (24%), greater representation of mixed/multiple ethnicities (32% vs. 16%), but lower proportion identifying as Asian/Asian British (17% vs 60%) [44]. This over-representation of participants identifying as Black or mixed race/multiple ethnic groups is suggestive of disparities in employment opportunities in addition to the structural inequalities discussed above. Structural racism limits employment options for ethnic or racialized minorities, leading to their overrepresentation in informal and often criminalized work, as has been evidenced in increased engagement in sex work in the USA among women in drug treatment identifying as African American and among trans-communities identifying as Black, American Indian, or Latina [20, 45].

## Strengths and Limitations

The lack of a probabilistic sample and small sample size limits inferences we can make to other sex workers. Generalizability is further restricted by the highly urban and ethnically diverse nature of the study boroughs, limiting comparability to similar urbanized areas. A key limitation is our inability to look at the effect of trans and gender-minority identity on police enforcement practices and emotional or sexual violence, due to the few people identifying as trans or non-binary recruited into our sample (< 10). We have included this population alongside their cis counterparts to include all participants’ responses and as past quantitative research has excluded trans and gender-minority sex workers. However, we acknowledge that this fails to capture the substantial impact of transphobia. International evidence points to increasing violence against trans sex workers by clients and police, made worse by police inaction to respond appropriately, highlighting the imperative to address transphobia within police, other institutions, and broader society and the need for targeted interventions to address the specific needs of gender-minority sex workers [5, 8, 46].

The study was not originally designed to assess differences in police enforcement or violence by ethnic/racial identity, which may have limited the ability to detect whether observed differences are statistically significant. Data show that Black women and those identifying as Gypsy, Roma, or Traveler experience disproportionate custodial remand and sentencing compared to Asian and white women [19, 47]. We recognize the risks of downplaying effects by collapsing ethnic/racial identity into a binary category and inability to document differential needs and experiences within ethnically or racially minoritized groups. However, this exploratory analysis was urgently needed, particularly in the context of limited UK-based research on the experiences of ethnically and racially minoritized sex workers. The sample size also precluded the measurement of the potential modifying effects of working in street settings on police enforcement; further research is needed to understand how these effects manifest for sex workers of different racial/ethnic identities and in different work settings. It is possible that migration status confounded the relationship between race/ethnicity and enforcement or violence. Previous research has suggested few differences in relation to sexual or physical violence or enforcement between migrant and UK-born sex workers working in off-street settings [48], although our and others’ qualitative research demonstrates how migrants are targeted by both police and immigration authorities [29, 49]. Reports of insecure residency were similarly distributed by ethnic/racial identity, and all multivariable models adjusted for migration status. However, we had difficulties in recruiting the minority of migrant women working in street-settings, prohibiting our ability to generalize findings to this population.

Our findings support the growing body of evidence showing that repressive policing disproportionately targets minoritized and marginalized sex workers, has a negative impact on sex workers’ health, and is linked to heightened risk of rape and emotional violence. The extensive police enforcement, violence, arrest, imprisonment, and extortion that land disproportionately on ethnic or racialized minority sex workers further support sex workers’ and human rights organizations’ calls to decriminalize sex work as a matter of racial justice as well as one of broader health and social justice [50]. Participants were most often enforced against for reasons other than sex work, in line with the qualitative study and international evidence which demonstrates how cis and trans women, who work on street and use drugs, are racially minoritized and/or migrants are disproportionately targeted by enforcement and denied access to justice [5, 46]. Decriminalization of sex work must therefore occur concurrently with decriminalizing drug possession and use, and addressing social exclusion and discrimination in relation to sex work, sexual and gender identity, housing, poverty, race, and ethnicity at a young age. Services are needed that can cater to these intersecting oppressions, particularly around preventing and addressing the consequences of sexual and emotional violence. Such interventions are currently inadequate and poorly evidenced [51, 52]. Mainstream and sex worker support services must be actively inclusive of and involve minoritized and marginalized sex workers in the development and delivery of services and receive full investment to do this. This is needed to ensure that sex workers who experience harms and neglect receive the resources and positive outcomes that they are entitled to. In light of these findings and mounting evidence of systemic racism, misogyny, and discriminatory practices within police and public services, we call upon regulatory bodies to evaluate services to ensure that government institutions appropriately respond to the diverse needs of sex workers rather than further entrenching inequalities and perpetuating criminalization, poverty, and stigma.
